# The Lasker-Bloomberg Public Service Award honors Michael J. Fox’s advocacy for Parkinson’s disease patients and research

**DOI:** 10.1172/JCI212281

**Published:** 2026-09-09

**Authors:** Dimitri Krainc

**Affiliations:** 1 Davee Department of Neurology, Northwestern University Feinberg School of Medicine, Chicago, Illinois, USA.; 2 President, American Neurological Association.

The Lasker Awards have long recognized individuals whose work has transformed medicine and improved human health. This year, the Lasker-Bloomberg Public Service Award is honoring Michael J. Fox (Figure 1). With this selection, the Lasker Foundation is affirming a broader truth: scientific progress depends not only upon brilliant investigators but also upon visionary advocates.

Michael is precisely such a visionary. His efforts show that celebrity can be used to make a meaningful impact. He did this by becoming an informed advocate who engaged scientists, policymakers, donors, and patients with equal authenticity. His credibility in this arena stemmed from decades of sustained commitment. The 2026 Lasker-Bloomberg Public Service Award recognizes an advocate whose steadfast commitment accelerated the global fight against Parkinson’s disease and inspired millions to believe that lives can be changed when hope, science, human resilience, and determination come together.

The public first got to know Michael’s unmistakable charisma on the screen. Whether captivating audiences as Alex P. Keaton in *Family Ties* or becoming a cinematic icon through his unforgettable portrayal of Marty McFly in the *Back to the Future* trilogy, he displayed an extraordinary gift for making his roles feel genuine and deeply human and connecting with audiences across generations. Few actors have achieved such enduring cultural significance while remaining so approachable and universally admired.

In 1991, at the age of just 29, Michael J. Fox was diagnosed with Parkinson’s disease. Parkinson’s is a progressive neurological disorder that gradually affects movement, balance, speech, and many aspects of daily living. Receiving such a diagnosis at the height of an acting career must have been devastating. For many, such news would understandably lead to retreat from public life. However, for several years, Michael carried this burden privately while continuing to work with professionalism and determination.

When Michael publicly disclosed his diagnosis in 1998, he did something remarkable by transforming his personal struggle into a mission that would benefit countless others. His openness helped dispel stigma surrounding Parkinson’s disease and other diseases, and it encouraged conversations with family, friends, and medical professionals that had too often been avoided. More importantly, he demonstrated that vulnerability could become a source of strength when it is used in the service of others.

## The Michael J. Fox Foundation’s transformative vision

The creation of The Michael J. Fox Foundation for Parkinson’s Research in 2000 marked the beginning of one of the most successful patient-driven scientific movements in modern medicine. It was built upon an ambitious, straightforward vision: to accelerate the development of better treatments and ultimately a cure for Parkinson’s disease (1). Michael insisted that urgency, collaboration, transparency, and innovation become the guiding principles driving the Foundation. To date, the Foundation has provided more than $3 billion in funding that has enabled the entire field to progress (1).

Over the past two decades, the Foundation has become the world’s leading nonprofit funder of Parkinson’s disease research. It has supported thousands of scientific projects across numerous countries to advance understanding of the disease’s biology, genetics, diagnosis, and treatment. By bringing together researchers, clinicians, patients, industry partners, and policymakers, the Foundation has helped break down barriers that traditionally slow scientific progress.

Early on, Michael J. Fox and the team he built at the Foundation understood something that many scientific organizations have since embraced: breakthroughs do not emerge solely from isolated laboratories. They arise through collaboration, open sharing of data, and a willingness to unite diverse expertise toward a common goal. Perhaps even more importantly, Michael recognized that patients themselves are indispensable partners in scientific discovery. Through large-scale research initiatives and patient engagement programs, individuals living with Parkinson’s disease have contributed valuable clinical information and biological samples that continue to inform and reshape researchers’ understanding of the disease. By encouraging patients to become active participants rather than passive recipients of care, he helped redefine the relationship between medicine and those it serves.

For Michael J. Fox, hope has always been inseparable from effort. Scientifically, that effort involves supporting scientific discovery, funding promising investigators, and encouraging clinical trials. It includes large-scale efforts like building partnerships among governments, universities, pharmaceutical companies, and patient communities. The Foundation’s research strategy communicates its belief that every experiment, every new technology, every young researcher, and every patient volunteer pushes us closer to a future in which Parkinson’s disease can be prevented, treated more effectively, or one day cured.

Equally remarkable is the trust Michael has inspired within the scientific community. Researchers often describe him as a genuine partner in advancing discovery. His foundation has earned widespread respect for supporting rigorous science, encouraging collaboration over competition, and emphasizing measurable outcomes. The trust the foundation earned in the process reflects years of thoughtful leadership and integrity.

The impact of the Foundation’s efforts extends beyond Parkinson’s disease alone. The Michael J. Fox Foundation has become a model for philanthropy-driven biomedical research, demonstrating how strategic investment and collaboration can accelerate innovation. Organizations addressing Alzheimer’s disease, ALS, multiple sclerosis, rare genetic disorders, and other neurological conditions have drawn inspiration from this approach. In this sense, Michael J. Fox’s advocacy has contributed to broader improvements in the way medical science is organized and supported.

## Facing adversity with strategic optimism

Beyond the scale of his foundation’s accomplishments, however, Michael J. Fox is also distinguished by the spirit in which they have been achieved. Throughout his journey, he has consistently chosen optimism as a deliberate and courageous response to his disability. He has spoken candidly about the physical challenges of Parkinson’s disease, acknowledging both the frustrations and uncertainties that accompany its progression. Yet he has refused to surrender his sense of humor, his curiosity, or his faith in human ingenuity.

At the heart of Michael J. Fox’s story lies an enduring lesson about the relationship between adversity and purpose. A defining feature of his character is that when faced with a devastating diagnosis, Michael asked, “What can I do?” Michael’s hope and the ambitious philosophy of his foundation have inspired countless families affected by Parkinson’s disease. Around the world, individuals confronting diagnoses have found reassurance in Michael’s example. They have seen someone who continues to advocate and contribute despite immense challenges.

## Recognizing a lifetime of tireless advocacy

Michael’s legacy and the legacy of his foundation will ultimately be measured not only in awards or public recognition but in discoveries yet to come. Every improved diagnostic tool, promising therapy, successful clinical trial, and future breakthrough in Parkinson’s disease research will carry, in some measure, the imprint of the movement he helped create. Generations of scientists who never met him will build upon research made possible through his advocacy. Patients not yet diagnosed will someday benefit from advances that trace their origins to his determination.

This Lasker Award therefore celebrates far more than achievement. It celebrates compassion transformed into action, resilience transformed into leadership, optimism transformed into scientific progress. For those living with Parkinson’s disease today, Michael J. Fox represents hope. For their families, he represents courage in the face of uncertainty. For researchers, he represents partnership, and for physicians, he represents an unwavering commitment to improving patient care. In the broadest sense, he represents the extraordinary impact that one determined individual can have when personal experience is harnessed for the common good.

As we celebrate Michael’s Lasker Award, we honor a humanitarian whose compassion has touched countless lives, and a leader whose vision continues to reshape the future of Parkinson’s research. His example teaches us that courage is not the absence of fear, resilience is not the denial of hardship, and hope is not passive optimism. Rather, each is expressed through purposeful action undertaken in the service of others.

Congratulations, Michael J. Fox. This distinguished recognition acknowledges a lifetime of extraordinary contributions to science, medicine, and humanity itself. Your legacy lives in every laboratory pursuing new discoveries, every patient participating in research, every family sustained by hope, and every future breakthrough that brings the world closer to curing Parkinson’s disease. For all that you have given, and for all that your work continues to inspire you have our immeasurable gratitude.

## Conflict of interest

DK is the founder of Vanqua Bio and Venture Partner at OrbiMed.

## Figures and Tables

**Figure 1:**
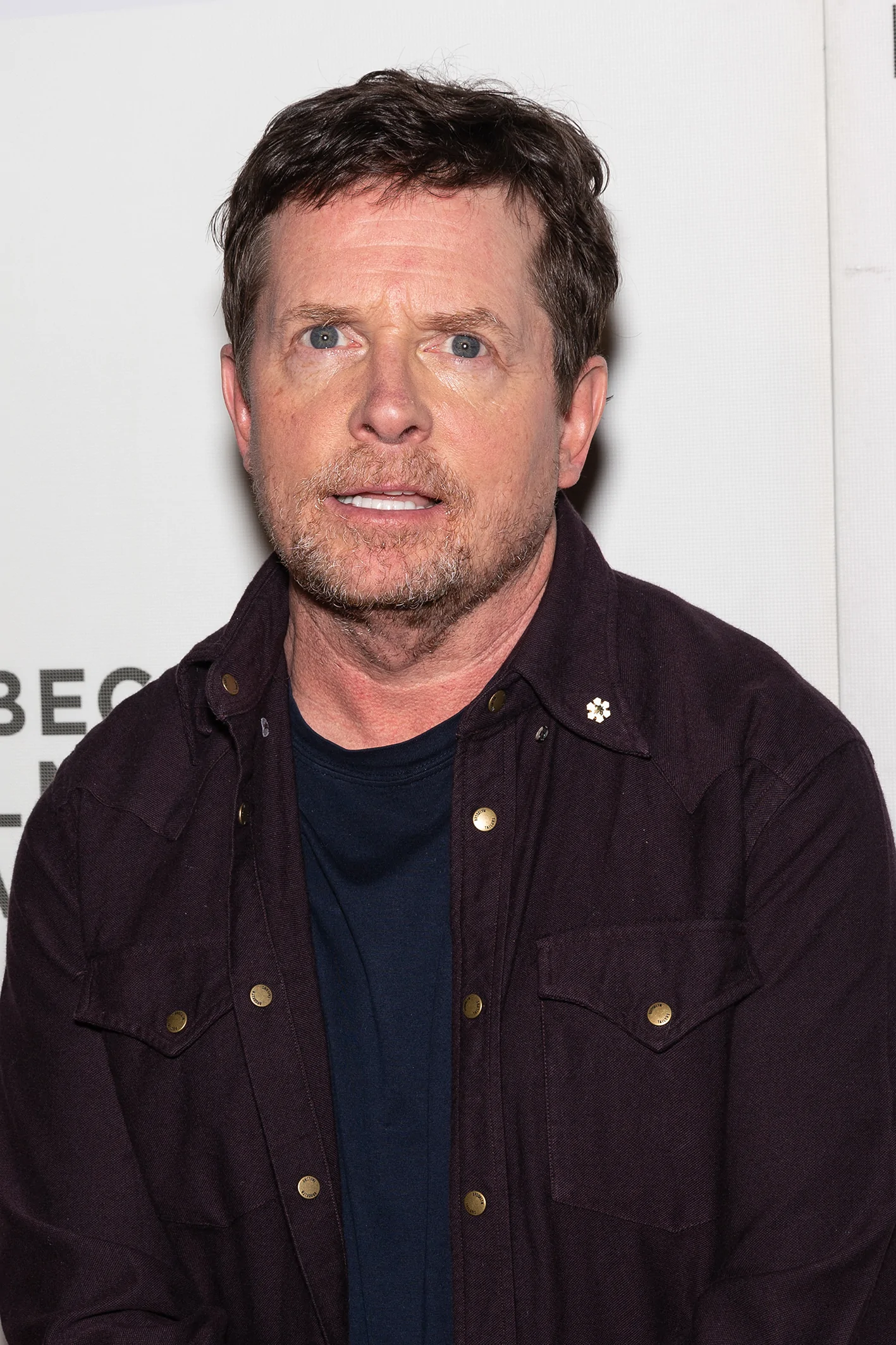
Michael J. Fox is the recipient of 2026’s Lasker-Bloomberg Public Service Award. The award recognizes Fox’s decades of advocacy, which has empowered patient voices and accelerated research to advance therapies and find a cure for Parkinson’s disease. Image credit: Ron Adar/Shutterstock.

## Version 1 09/09/2026

Online First

## Version 2 09/15/2026

Electronic publication
